# Cognitive Bias Modification Reduces Social Anxiety Symptoms in Socially Anxious Adolescents with Mild Intellectual Disabilities: A Randomized Controlled Trial

**DOI:** 10.1007/s10803-018-3579-9

**Published:** 2018-04-21

**Authors:** Anke M. Klein, Elske Salemink, Eva de Hullu, Esther Houtkamp, Marlissa Papa, Mariët van der Molen

**Affiliations:** 1 Clinical, Neuro, and Developmental Psychology, VU Amsterdam, Amsterdam, The Netherlands; 2 Developmental Psychology, University of Amsterdam, Nieuwe Achtergracht 129B, Amsterdam, The Netherlands; 3 Clinical Psychology, Open University, Heerlen, The Netherlands

**Keywords:** Cognitive bias modification, Interpretation bias, Content-specificity, Social anxiety, Mild intellectual disability

## Abstract

The goal of this study was to examine the effects of Cognitive Bias Modification training for Interpretation (CBM-I) in socially anxious adolescents with Mild Intellectual Disabilities (MID). A total of 69 socially anxious adolescents with MID were randomly assigned to either a positive or a neutral control-CMB-I-training. Training included five sessions in a 3-week period, and each session consisted of 40 training items. Adolescents in the positive training group showed a significant reduction in negative interpretation bias on the two interpretation bias tasks after training compared to adolescents in the control-training group. Furthermore, in contrast to the control-training group, adolescents in the positive training reported a significant reduction of their social anxiety symptoms 10 weeks post-training.

## Introduction

Social anxiety disorder is highly prevalent in adolescents and is reported as one of the most common forms of social distress in this population, also in adolescents with Mild Intellectual Disabilities (MID; Dekker and Koot 2003; Kessler et al. 2005). Social anxiety disorder often develops during adolescence and is marked by the persistent fear of social or performance situations (American Psychiatric Association 2013). Although treatments for child- and adolescent anxiety have shown good efficacy, at least 40% of children and adolescents continue to have a diagnosis after treatment (James et al. 2013). In particular, research suggests that socially anxious children and adolescents have the poorest outcomes following treatment when compared to other anxiety disorders, and they are only half as likely to remit as children and adolescents with other anxiety disorders, resulting in lifelong impairment (e.g., Hudson et al. 2010). Moreover, even though the prevalence rates of social anxiety are comparable in adolescents with MID (Dekker and Koot 2003), studies related to the treatment of anxiety disorders in adolescents with MID are scarce. However, the general conclusion is that treatments are even less effective in individuals with MID compared to individuals with an average IQ (see also Dagnan and Jahoda 2006). Generally, therapies like cognitive behavioral therapy, are often too complex and demanding for people with MID (De Wit et al. 2012). To develop more effective treatments for social anxiety disorder appropriate for adolescents with MID, it is important to find theoretical and practical innovations that might take current treatments into new directions.

Several underlying processes have been defined as maintaining and possibly causing anxiety disorders, including cognitive processes (for a review, see Mathews and MacLeod 2005). According to cognitive theories of anxiety disorders (e.g., Beck et al. 1985), socially anxious adults and children have anxiety-related schemata that direct processing resources towards threat-relevant information resulting in cognitive biases related to attention, interpretation and memory (e.g., Clark and Wells 1995; Rapee and Heimberg 1997). Numerous studies found evidence for the existence of cognitive biases, such as attention bias and interpretation biases, in anxious adolescents (for an overview, see Hadwin and Field 2010). A recent meta-analysis with regard to attention bias found a small positive association between anxiety and attention bias (*d* = 0.21; Dudeney et al. 2015). Furthermore, they found a moderating effect of age; the relation between attention bias and anxiety increased with age. A meta-analysis with regard to interpretation bias found a medium positive association between anxiety and interpretation bias (*d* = 0.62; Stuijfzand et al. in press). Furthermore, they found a moderating effect of the content of ambiguous scenarios in their meta-analysis; the relation between anxiety and interpretation bias was stronger when the ambiguous scenarios matched the anxiety subtype under investigation. However, the authors pointed out that this effect was mainly driven by studies that focused on social anxiety.

Even though there are numerous studies with regard to attention- and interpretation bias in typically developing children and adolescents, to the best of our knowledge, no studies were published on the relation between anxiety and attention bias in adolescents with MID, and only a few published studies that found evidence for the relation between anxiety and interpretation biases in adolescents with MID (Houtkamp et al. 2017; Van der Molen and Salemink 2016), For example, Van der Molen and Salemink (2016) assessed interpretation bias using ambiguous scenarios in adolescents with varying levels of IQ (*min* = 55, *max* = 129, *Mean* = 85). Results showed a medium positive significant relation between anxiety and interpretation bias, where higher levels of anxiety are associated with stronger threat-related interpretations, while IQ did not moderate this effect.

To investigate the role of threat-related interpretation biases in adolescents with MID in more detail and the potential to alleviate symptoms by reducing biases, the current study focused on Cognitive Bias Modification for Interpretation (CBM-I) training. In CBM-I, participants learn to restructure the way they interpret ambiguous related situations with the goal to reduce threat-related interpretation biases and reduce levels of anxiety symptoms (for meta-analyses in adolescents, see Cristea et al. 2015b; Krebset al. in press). When the first meta-analysis on the effects of CBM for interpretation and attention in adolescents came out (Cristea et al. 2015b), the conclusions were rather unfavorable towards CBM: CBM did not affect anxiety. This conclusion shed doubt on the clinical relevance of CBM techniques, while noticing that most studies were suboptimal. More recently, however, Krebs et al. (in press) conducted another meta-analysis, now specifically focusing on the effects of CBM-I, and found a moderate effect on negative- and positive interpretation bias (negative: *g* = − 0.70/positive: *g* = − 0.52), and a small, but significant effect on anxiety directly following training (*g* = − 0.17). One of the reasons for these different findings is that Krebs et al. (in press) only included studies that focused on CBM-I. This might be an advantage, as there is some evidence that that CBM for interpretation bias is more effective than CBM for attentional bias (see also Cristea et al. 2015a; Lau 2015). Furthermore, a recent study by Grafton et al. (2017) re-analyzed the meta-analysis by Cristea et al. (2015b) showing that indeed CBM procedures do not always have an impact on mental health concerns. However, this is only correct when the cognitive bias has not changed during the CBM training. When CBM procedures successfully modify cognitive biases, this often results in a significant reduction in mental health concerns. In sum, several studies see the potential of CBM-I, but also acknowledge the fact that more research is needed and that several improvements are to be made (see also Krebs et al. in press). Furthermore, to the best of our knowledge, there are no published studies on the effects of CBM-I in adolescents with MID. However, CBM-I is a promising training for adolescents with MID, as the training does, compared to regular therapies, not appeal on reflection, meta-cognition, or other, for adolescents with MID, cognitive too demanding exercises.

In order to use CBM-I in adolescents with MID and to make improvements in our CBM-I procedures in general, it is important to focus on the details of the different studies that have been conducted thus far and on the recommendation made by these studies. Overall, interpretation bias training has proven to be capable of reducing biases in adolescents (e.g., Salemink and Wiers 2011) with long-term effects (De Hullu et al. 2017). Effects on anxiety (e.g., Reuland and Teachman 2014) and stress appraisal (e.g., Lau et al. 2012) have been less robust. CBM-I has shown to be specifically effective in adolescents with lower levels of cognitive control or working memory capacity (Salemink and Wiers 2012). As precisely adolescents with MID have difficulties in making use of their working memory (Van der Molen et al. 2010), CBM-I might be particularly relevant here as an alternative approach in treatment. Furthermore, the largest effect of CBM (on bias and symptoms) was found when training was performed within the school setting rather than, for example, via internet (Cristea et al. 2015b). Several ways forward have been formulated to improve CBM research. Hirsch et al. (2016), for example, suggested that future studies should focus on disorders in which the negative resolving of ambiguity is a key aspect, such as social anxiety disorder, while paying attention that the used scenarios are idiosyncratic and address the disorder-specific ambiguity at the same time (see also Klein et al. 2015).

Many of the details and recommendations listed above were addressed in the current study that tested the efficacy of a CBM training in adolescents with MID. This study (1) focused solely on interpretation, (2) included five training sessions, and (3) used carefully selected stimuli focusing on social anxiety, (4) in a classroom setting. Based on earlier CBM-I studies in anxious adolescents with an average IQ, we hypothesized that adolescents in the positive training group would show significant reductions in interpretation biases and self-reported social anxiety after training, which we did not expect for adolescents in the neutral control-group.

## Methods

### Participants

**Fig. 1:**
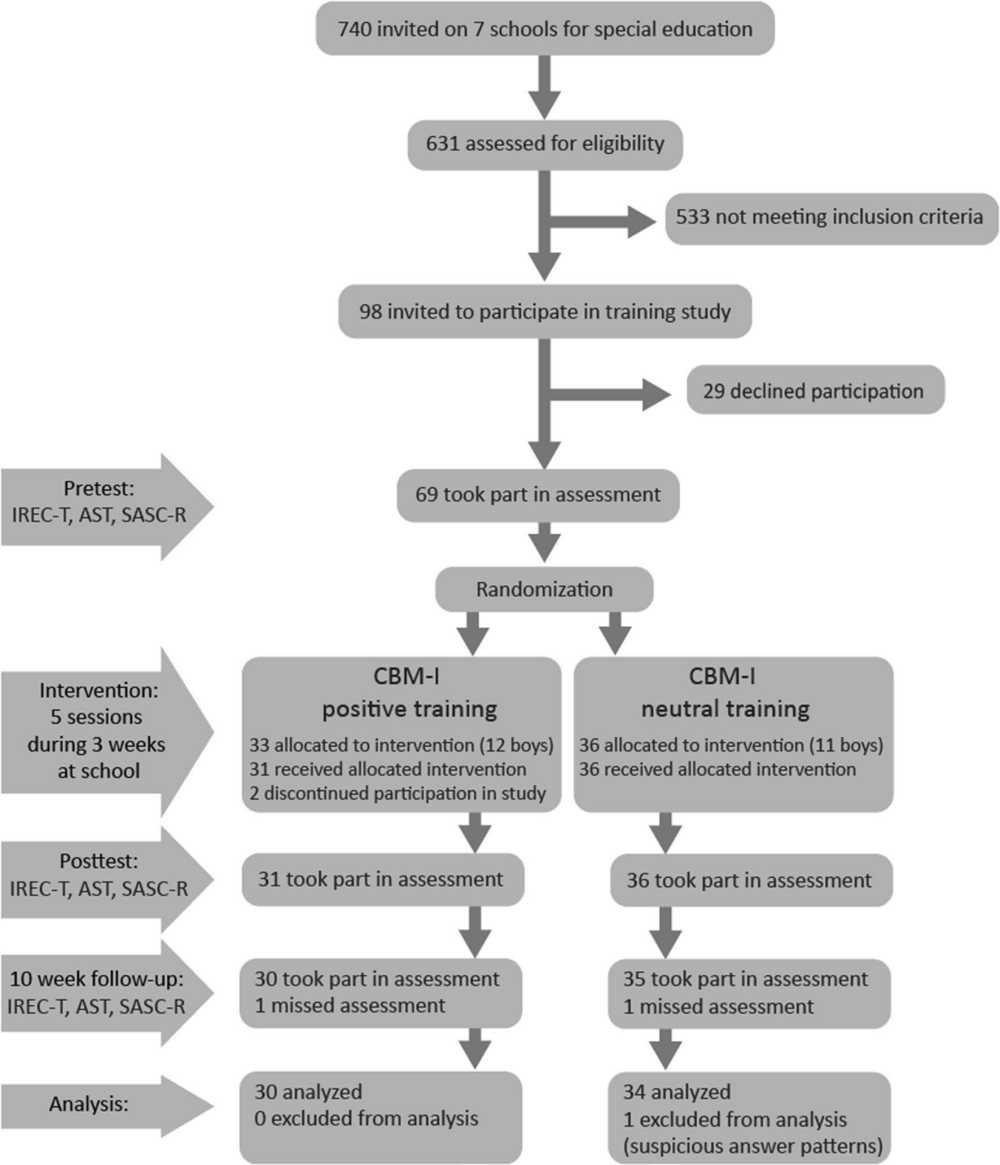
Participant flow

A sample of 740 adolescents was recruited from seven secondary schools for students with mild to borderline intellectual disabilities in the Netherlands. These schools include students with MID with IQ-scores varying between 60 and 85 in combination with more than 3 years of learning deficits (Van Rijswijk and Kool 2002). After passive consent had been granted by the adolescents and their parents (Sept–Oct 2015), a total of 631 adolescents participated in the screening part of this study (Oct–Dec 2015). All adolescents were between 12 and 18 years old (*M* = 14.4, *SD* = 1.5). Directly following screening, adolescents scoring above the clinical cut-off score (Sum Score = 8) on the social phobia subscale of the SCARED-NL-71 (Bodden et al. 2009) during the screening (min = 0, max = 18, *M* = 4.54, *SD* = 3.66), were selected to attend the training. A total of 98 adolescents scored above this cut-off score (min = 9, max = 18, *M* = 10.88, *SD* = 2.11). Internal consistency of the social phobia subscale in the current sample (*α* = 0.82) was good, and comparable to the original psychometric study of Bodden et al. (2009; *α* = 0.85). Selected adolescents (and their parents) received an information letter with an invitation to participate in the study. The letter explained the study in detail and both the adolescent and their parents had to give approval to participate in the training. After active consent was signed, adolescents were randomly allocated to either the positive training or the neutral control-training. In total, 69 adolescents participated in the training; 33 adolescents received the positive training and 36 adolescents the neutral control-training (see Fig. 1 for the participant flow; Feb–May 2016). Participant numbers were allocated before the screening phase started. Allocation to training type was based on participant number; even numbers received the neutral training, uneven numbers received the positive training (simple randomization). The research assistants on the project enrolled the participants and assigned the participants to the intervention. A power calculation with G*Power 3.1.2 showed that a group of at least 26 adolescents per condition was needed to yield statistical power of 1 − β = 0.80 at *p* = .05 (two-tailed) for a medium effect size (*F* = 0.20). Due to possible dropout, we aimed to include approximately 30–35 adolescents per group.

The ethics committee of the University of Amsterdam, approved the study. This clinical trial was registered in ISRCTN registry with number ISRCTN73367465 and followed the CONSORT guidelines. The materials reported here are part of a larger community-based project on adolescent anxiety in MID. That is, participants also completed likability questions and a general measure of interpretation bias during the screening phase of the project (Houtkamp et al. 2017; Klein et al. 2017). During the training phase, they also completed several other questionnaires and tasks (secondary outcomes) that were not the primary aim of the current study. In the current study, we only report the primary outcomes of the study. A full list of materials and tasks is available by contacting the corresponding author.^1^

### Instruments

#### Cognitive Bias Modification Training

To modify interpretation bias, adolescents completed a training program of five sessions in a 3-week period (see also Salemink et al. 2009). Each session consisted of 40 ambiguous scenarios. Each scenario consisted of three short sentences, with a word in the last sentence missing. We created two different versions of the training task, a positive training and a neutral control-training (see Table 1 for a sample of each). In the positive training, all ambiguous scenarios were related to social situations and the word fragment made the story end positively. In the control training, all ambiguous scenarios were non-emotional and the word fragment made the story end in a neutral non-emotional way that was irrelevant to anxiety.

**Table 1:** Sample stories of the CBM-I training, and both interpretation tasks (IREC-T/AST)

Sample scenarios training	
Positive training scenario 1. You give a presentation in your class. Everyone is listening. A few girls are smiling. You think they … your presentation L.KE 2. LIKE 3. Do the girls like your presentation? a. Response participant = YES (correct answer), then: Indeed, they very much like your presentation b. Response participant = NO, then: they like your presentation	Neutral control-training scenario 1. You are at the bakery. Everything is looking yummy. You choose an apple beignet. It is still… W.RM 2. WARM 3. Is the apple beignet cold? a. Response participant = YES, then: The apple beignet is still warm b. Response participant = NO (correct answer), then: Indeed, the apple beignet is still warm

Sample interpretation recognition task (IREC-T) scenario	
A. You walk on your own down the street and see a group of classmates talking. When you pass the group, they start laughing	Did you walk with a friend down the street? Yes/no
B. You walk on your own down the street and see a group of classmates talking. When you pass the group, they start laughing What is the chance that they start laughing because they find you strange?	The chance is… 1. Very small 2. Small 3. Great 4. Very great

Sample ambiguous scenarios task (AST) scenario	
Birthday Today it is your grandmother’s birthday. You give your grandmother a present. Everybody is watching when grandma opens the present. Then all of a sudden someone laughs really loud…	1. Everybody thinks the present is stupid 2. You hope it is not your present they are laughing about 3. Your uncle is making jokes with your cousin 4. You have a funny present and everyone likes it

A set of 200 scenarios was used. The scenarios of this set were adapted for adolescents with MID from existing materials (Creswell et al. 2005; De Voogd et al. 2018; Klein et al. 2015; Salemink et al. 2009), or created by the authors in collaboration with practitioners. Four versions of all scenarios were created so that the scenarios matched the gender of the participant and so that there was one set of 100 scenarios with neutral scenarios and one set of 100 scenarios with positive scenarios. Across training and within each training session, the scenarios were randomized for all adolescents. All scenarios were shown twice in a fixed order, so that the time between appearances of the same scenario was as long as possible. In addition to reading the scenarios, the scenarios were simultaneously presented through audio, so that the adolescent was able to hear and read the scenarios during the task (see also Blackwell et al. 2015).

First, the participants were asked to read each scenario and imagine themselves as the central character. After reading a scenario, the participant pressed the space bar and the missing final word appeared on the screen with one letter missing. The participant’s task was to fill in this missing letter, after which they received feedback by reading the correct response. Third, the participant was asked to answer ‘yes’ or ‘no’ to a question that measured comprehension of the story. This comprehension question reinforced either the positive or neutral interpretation. Directly following the answer, participants received feedback regarding their response to this comprehension question. In case the participant gave the correct answer, the participant received the feedback ‘indeed’ followed by the correct interpretation. In case the participant gave the wrong answer, he/she simply received the correct interpretation. See also Table 1 for a sample story including the feedback.

#### Interpretation Bias

To test near transfer and far transfer of learning, we used two tasks to measure interpretation bias. First, we used the Interpretation Recognition Task (IREC-T; Houtkamp et al. 2017; Salemink and Van den Hout 2010) in which participants completed 8 ambiguous scenarios. The IREC-T uses easy words and short sentences, and is very comparable to the training task (see also Houtkamp et al. 2017). The 16 scenarios, all related to social situations, were used to randomly create two sets of 8 scenarios that were distributed randomly across pre-training and post-training assessments. The adolescents were randomly assigned to perform one set before training and one set after training. During the 10-week-follow-up, adolescents performed the first set again. Internal consistency was sufficient for all three measurements (T1: α = 0.78, T2: α = 0.78, T3: α = 0.78).

Second, we used the AST (e.g., Klein et al. 2014, 2015). The AST also measures interpretation bias, but has a different task structure than the training task and the IREC-T. We decided to include this second interpretation task, as we wanted to test the generalization of the training effect to an interpretation task that is less similar to the training. The AST in this study consisted of 16 multiple-choice social-threat related ambiguous scenarios (See Table 1 for a sample story). The set of 16 scenarios was adapted (easier words/shorter sentences) and translated into Dutch from existing materials (see also Klein et al. 2014, 2015). These 16 scenarios were then used to randomly create two sets of 8 scenarios each. The adolescents were randomly assigned to perform one set before training and one set after training. During the 10-week-follow-up, adolescents performed the first set again. Internal consistency was sufficient for all three measurements (T1: α = 0.68, T2: α = 0.65, T3: α = 0.74).

#### Social Anxiety Scale for Children—Revised (Greca and Stone 1993)

The SASC-R is a self-report questionnaire measuring symptoms of social anxiety with 18 items on a five-point rating scale ranging from ‘not at all’ to ‘all the time’. To make sure that the adolescents with MID understood the questions correctly, some possibly difficult words were explained in brackets. Internal consistency in the current study/sample was excellent for all three measurements (T1: α = 0.88; T2: α = 0.90; T3: α = 0.91).

### Training Procedure

First, adolescents were invited to an assessment session in which they performed the IREC-T, the AST and the SASC-R individually in a testing room at their own school. They then received five training sessions in 3 weeks’ time. To provide an optimal training setting, one-on-one instruction was provided and all sessions took place individually, within the school setting. Each training session, performed on a school computer, took about 20–30 min and participants were able to take short breaks after every 10 scenarios. Short and long-term effects were tested by assessing bias and anxiety both at posttest and 10-week-follow-up: directly following the last training session, and during the 10-week-follow-up, the participants performed the assessment session including the IREC-T, the AST and the SASC-R again. A trained Master’s level student in Developmental Psychology accompanied all session. Participants received a voucher worth 5€ as a reward after finishing the entire procedure.

## Results

### Descriptives

Demographics and characteristics of the positive and neutral control-training group are presented in Table 2. There were no significant group differences pre-training in age, gender, interpretation biases, and social anxiety scores (all *p*’s > .1).

**Table 2:** Demographics and pre-training characteristics of the participants

	Positive training group	Neutral control-training group
	M (SD)	M (SD)
Age	14.4 (1.6)	14.4 (1.5)
Social anxiety	3.0 (0.9)	1.7 (0.9)
IREC-T	2.5 (0.6)	2.4 (0.6)
AST	2.6 (0.6)	2.9 (0.5)

*IREC-T* interpretation recognition task, *AST* ambiguous scenarios task

### Near Transfer of Training Effect on Interpretation Bias: IREC-T

**Fig. 2:**
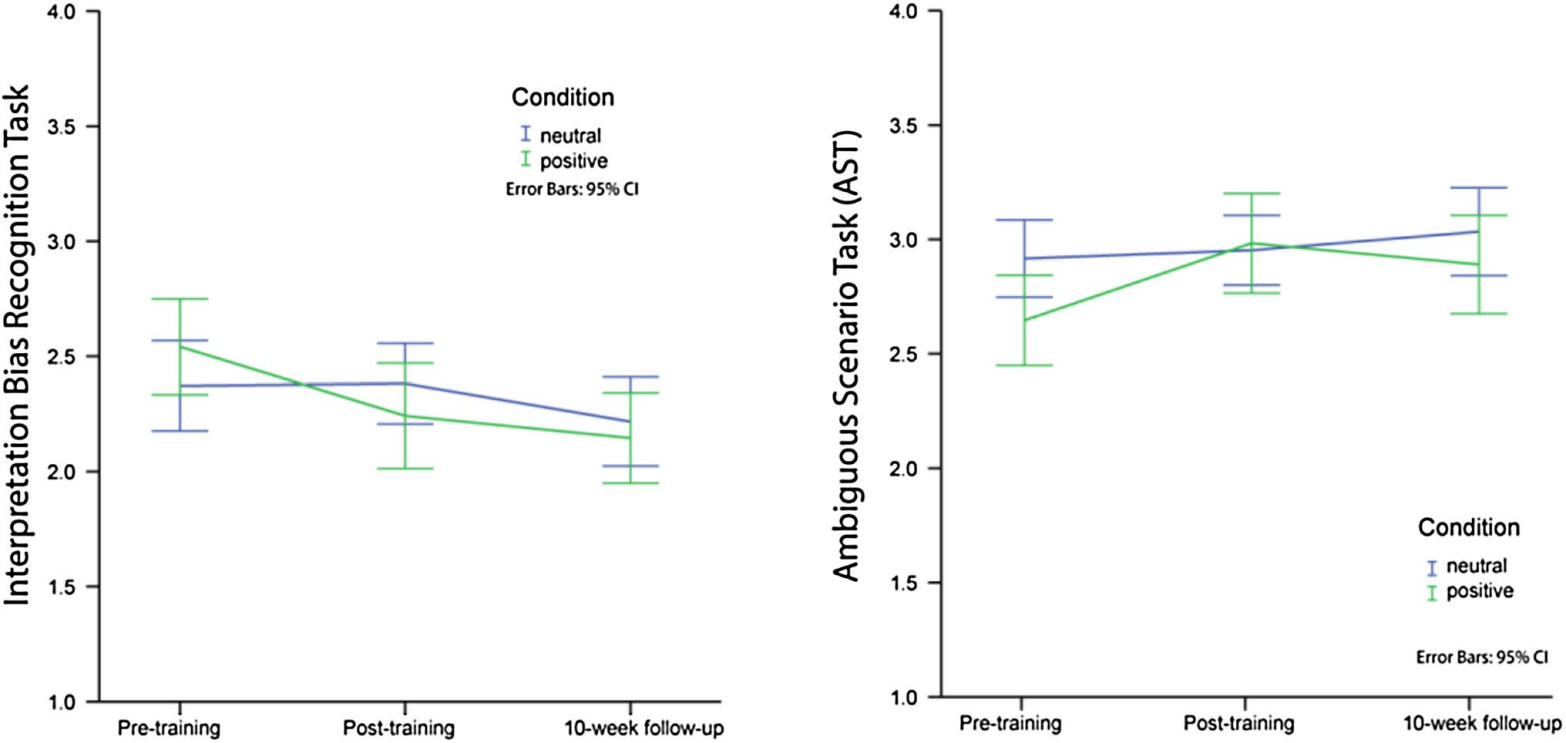
Effects of training on both interpretation tasks: IREC-T and the AST

To examine near transfer effect of training as measured with the IREC-T, we conducted a repeated-measures ANOVA with Training Group (positive/control) and Version Set (Version1/2) as between-subjects factors, and Time (pre-training/post-training/10-week-follow-up) as a within-subjects factor (see Fig. 2). The analysis revealed a significant main effect of Time *F*(2, 59) = 14.22, *p* < .001, *η*^*2*^ = 0.33, and this main effect was qualified by a significant interaction of Training Group x Time, *F*(2, 59) = 5.02 *p* = .010, *η*^*2*^ = 0.15. Tests of within contrasts revealed a significant interaction effect between pre-training and post-training *F*(1, 60) = 8.30, *p* = .006, *η*^*2*^ = 0.12, and between pre-training and 10-week-follow-up *F*(1, 60) = 6.69, *p* = .012, *η*^*2*^ = 0.10, but no significant interaction effect between post-training and 10-week-follow-up *F*(1, 60) = 0.25, *p* > .11, *η*^*2*^ < 0.01. As expected, additional paired-samples t-tests revealed a significant reduction in interpretation biases in the positive group from pre- to post-training, *t*(30) = 3.33, *p* = .002 and from pre-training to 10-week-follow-up *t*(30) = 5.54, *p* < .00, and no significant reductions in the control group from pre- to post-training: *t*(34) = − 0.19, *p* > .1. However, unexpectedly, there was a small but significant reduction in the control-training group from pre-training to 10-week-follow-up: *t*(33) = 2.17, *p* = .037. Thus, in the positive training group, the negative interpretation bias decreased during the training phase, and remained stable up to 10-week follow-up. The control-training group showed no reduction in negative interpretation bias during the training phase, but a small and significant decrease in bias from post-training to 10-week-follow-up.

### Far Transfer of Training Effect on Interpretation Bias: AST

To examine far transfer effect of training as measured with the AST, we repeated the above-mentioned analysis, but with the AST as the dependent variable (see Fig. 2). The analysis revealed a significant main effect of Time *F*(2, 58) = 5.45, *p* = .007, *η*^*2*^ = 0.16, and this main effect was again qualified by a significant interaction of training group × time for the social threat scenarios, *F*(2, 58) = 4.53, *p* = .015, *η*^*2*^ = 0.14. Tests of within subject contrasts revealed a significant interaction effect between time points pre-training and post-training *F*(1, 59) = 9.01, *p* = .004, *η*^*2*^ = 0.13, but no significant interaction between pre-training and 10-week follow up *F*(1, 59) = 1.61, *p* > .1, *η*^*2*^ = 0.03. Furthermore, there was a significant interaction effect between post-training and 10-week follow-up *F*(1, 59) = 4.33, *p* = .04, *η*^*2*^ = 0.07, but in the opposite direction as expected. As expected, additional paired samples t-tests revealed that the interpretations became significantly more positive in the positive group from pre- to post-training, *t*(30) = − 4.16, *p* < .001, and no significant increase in the control group, *t*(33) = − .11, *p* > .1. No significant increase from post-training to 10-week-follow-up were observed for both training groups (positive group, *t*(29) = 1.67, *p* > .1; control group, *t*(32) = − 1.4, *p* > .1). Thus, in the positive training group, positive interpretations increased significantly during the training phase, while decreasing slightly but non-significantly from post-training to 10-week follow-up. The control-training group showed a slight but non-significant increase in positive interpretations during the training phase, and again a slight, but not significant, increase in positive interpretations from post-training to 10-week-follow-up explaining the reversed interaction effect.

### Effects of Training on Self-Reported Social Anxiety

**Fig. 3:**
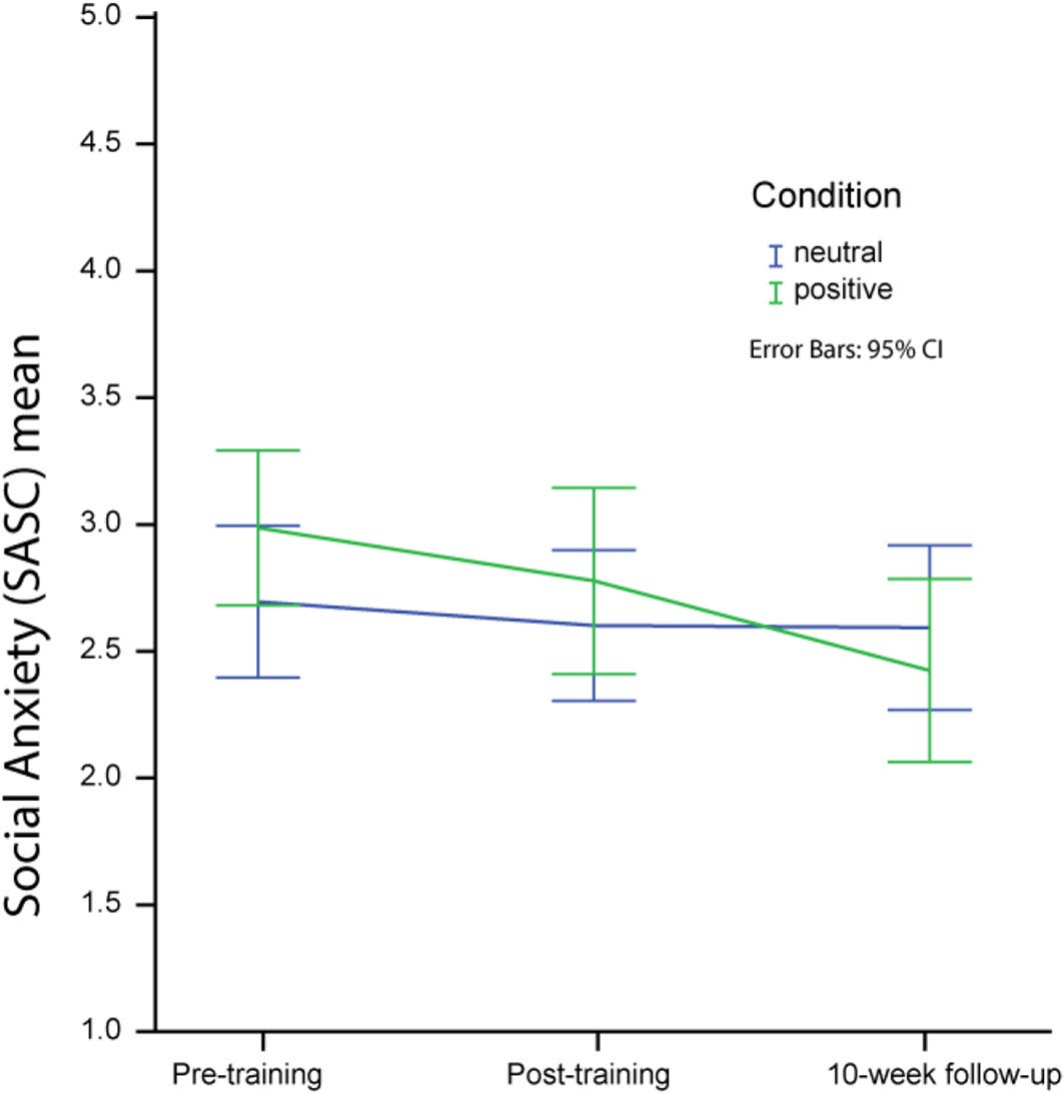
Effects of training on Social Anxiety (SASC-R) over time

To analyze the hypothesized effect of training on adolescent’s self-reported social anxiety as measured with the SASC-R, we conducted a repeated-measures ANOVA with Training Group (positive/control) as between-subjects factor, and Time (pre-training/post-training/10-week-follow-up) as a within-subjects factor (see Fig. 3). The analysis revealed a significant main effect of Time *F*(2, 60) = 7.24, *p* = .002, *η*^*2*^ = 0.19, which was qualified by a significant interaction of time × group *F*(2, 60) = 3.51, *p* = .036, *η*^*2*^ = 0.11. Tests of within subject—contrasts revealed no significant interaction effect between pre-training and post-training *F*(1, 61) = 1.35, *p* > .1, *η*^*2*^ = 0.05, but a significant interaction effect between pre-training and 10-week follow-up *F*(1, 61) = 6.84, *p* = .011, *η*^*2*^ = 0.10 and between post-training and 10-week follow-up *F*(1, 61) = 1.20, *p* = .047, *η*^*2*^ = 0.06. As expected, additional paired samples t-tests revealed a significant reduction in self-reported social anxiety in the positive condition from pre-training to 10-week-follow-up and from post-training to 10-week-follow-up, (pre-training to 10-week-follow-up: *t*(29) = 4.30, *p* < .001; post-training to 10-week-follow-up: *t*(29) = 3.41, *p* = .002), and no significant reduction in the control condition (pre-training to 10-week-follow-up: *t*(33) = 4.82, *p* > .1; post-training to 10-week follow-up: *t*(32) = 0.59, *p* > .1). Thus, in the positive training group, reduction in social anxiety symptoms was not reported directly following treatment, but did reduce significantly in the period from post- to 10-weeks post training. This significant reduction was absent in the control-training group.

## Discussion

The goal of this study was to examine the effects of a Cognitive Bias Modification training for Interpretation (CBM-I) in socially anxious adolescents with MID. As predicted, we found that adolescents in the positive training group showed significant reductions in interpretation bias after training, which was not found in the control-training group. We found evidence for an effect of near and far transfer of learning; Adolescents in the positive training group showed a significant reduction on the IREC-T, which was very similar to the training task, as well as on the AST, which measured interpretation bias using a different format. Furthermore, as expected, we found a significant reduction on self-reported social anxiety in the positive group, but only after 10-weeks-follow-up and not directly following training. To the best of our knowledge, this is the first study in adolescents with MID that shows that interpretation biases and social anxiety can be reduced in highly socially anxious participants by a CBM training that is characterized by content-specific training materials, with one-on-one instruction within a school setting, and multiple training sessions. These results are in line with other studies on adolescent CBM-I (e.g., Hirsch et al. 2016) and reflect recent meta-analyses (Cristea et al. 2015a, b; Grafton et al. 2017; Krebs et al. in press; for a comment on see Lau 2015) that suggest that CBM-I in adolescents might be effective under specific circumstances.

As learning difficulties are a central aspect of MID, we think it is remarkable that we were able to modify cognitive habits through a simple one-on-one CBM training paradigm provided by trained research assistants. Even though clearly more research is needed, this study indicates that a relatively simple technique can be used to lower anxiety symptoms in adolescents with MID. Moreover, CBM-I may be particularly beneficial, as previous studies show that regular treatments might be less effective to individuals with MID (see also Dagnan and Jahoda 2006). Our results are in line with the findings of Salemink and Wiers (2012) who found that CBM-I was more effective in adolescents with lower levels of cognitive control or working memory capacity. As precisely adolescents with MID have difficulties in making use of their working memory (Van der Molen et al. 2010), our findings might implicate that cognitive bias modification (CBM) procedures are suitable for populations with MID. It might be interesting for future studies to examine the effect of a combined therapy including regular treatments such as cognitive behavioral therapy (CBT) and CBM procedures. It might well be that combining CBT with CBM increases the efficacy of CBT in adolescents with MID as adolescents with MID might simply need more practice than in standard CBT treatments. By combining CBT with CBM, adolescents with MID get more help in practicing new, helpful thoughts over and over again, without the support of a therapist.

Whereas we found training effects on both interpretation bias measures, it should be noted that only the effect of near transfer of learning (IREC-T) was maintained in the long term but not the effect of far learning (AST). These results might indicate that adolescents did learn how the training worked, they ‘learned the trick’, but that they potentially need more practice to also positively interpret ambiguity in different situations on the longer term. The fact that the effects on the AST disappeared on the longer term might indicate that booster sessions may be necessary to sustain the training effect. Moreover, the effects on self-reported social anxiety were only visible on the longer term, and not directly following training. In line with cognitive theory, anxiety would be reduced only after repeated exposure to ambiguous situations in which more positive interpretations are applied. Since self-report measures of anxiety probe experienced recently anxiety, effects could only be expected after the positive interpretation style has been applied over a longer period of time (see also Harmer et al. 2009). These results show that it is important to include a follow-up measurement. However, this does not match well with the results on the AST, where we did not find a significant effect from pre- to 10-weeks follow-up. One would expect that applying a new interpretation style implies that the effect on the AST should have still been there at 10-week follow-up. We have no clear explanation as to why the effects of the AST were not maintained at 10-week follow-up. It could be that the AST taps into a different interpretation process than what has been learned during training (see also Klein 2016), but this should clearly be investigated in a larger follow-up trial including booster sessions and a longer follow-up measurement.

A few limitations of our study should be mentioned. First, we included adolescents who scored above the clinical cut-off score on social anxiety, but we did not administer a diagnostic interview to find out if adolescents had a clinical diagnosis or not. Second, we were unfortunately not able to measure IQ during our study due to time limitations. In addition, we were also not allowed to collect the IQ reports from the schools, due to privacy restrictions. We therefore cannot say anything about the relation between the CBM-I outcomes and IQ. A recent study of Van der Molen and Salemink (2016) studied interpretation bias in a sample of adolescents with varying IQ scores ranging in the MID range but also in the normal range, and they did not find an effect of IQ on the relation between interpretation bias and social anxiety. Also, the results of our study are comparable to samples including adolescents with an average IQ (e.g., Klein et al. 2015). Nevertheless, more research is needed that includes IQ as a moderator in the relation between anxiety and interpretation bias, and the effect of CBM-I before clear conclusions can be drawn. Third, we included a 10-week follow-up measurement, but we did not include a follow-up after a longer period, such as 6- or 12 months. Therefore, we cannot make predictions about the long-term effect of CBM-I on interpretation bias and anxiety symptoms in this population. Finally, our sample was relatively small and results showed small effects. Large-scale (clinical) trials including are needed before firm conclusions can be drawn with regard to the (clinical) implications of this CBM-I training.

In conclusion, this is the first study that examined the possible benefits of a CBM-I training on the decrease of anxiety symptoms in an understudied and often overlooked sample, namely adolescents with MID. In the current study, we addressed many of the theoretical and methodological limitations that were noted in recent meta-analyses of CBM (Cristea et al. 2015b) and CBM-I (Grafton et al. 2017; Krebs et al. in press). In a small but carefully designed randomized controlled trial, we showed that it is possible to modify interpretation biases in adolescents with mild intellectual disabilities with a CBM-I training, using social-anxiety specific stimuli, delivered in a controlled environment at school. This simple intervention resulted in a decrease in negative interpretations and in social anxiety 10-weeks following training. Even though clearly more research is needed, this first study in adolescents with MID shows the potential benefits of CBM-I for this population. CBM-I might be particularly beneficial combined with regular CBT, as adolescents with MID are able to practice many possible situations in a standardized environment without the need for a therapist.

## Footnotes

1 Originally, we planned to also examine social anxiety symptoms as reported by parents and teachers (SASC-R parent/teacher version and SCARED social phobia parent/teacher version), and self-reported social anxiety of the parent and teacher (adult version of the SCARED social phobia subscale) as a primary outcome of our study. Unfortunately, we received very minimal response. Only a few questionnaires were returned from parents and teachers, therefore these variables were excluded from this report. In order to compare the scores of parent, teacher and child, we also administered the SCARED social phobia subscale to the children. Since parent and teacher reports lack reliability, the child scores were also not reported in this paper. Note that a repeated-measures ANOVA showed a marginally significant Condition x Time interaction effect in the same direction as the SASC-R* F*(2, 60)=2.55,* p* = .087,* η*^2^ = .78
